# The regulatory role of m^6^A modification in the maintenance and differentiation of embryonic stem cells

**DOI:** 10.1016/j.gendis.2023.101199

**Published:** 2023-12-19

**Authors:** Jin Zhang, Lingling Tong, Yuchen Liu, Xiang Li, Jiayi Wang, Ruoxin Lin, Ziyu Zhou, Yunbing Chen, Yanxi Chen, Yirong Liu, Di Chen

**Affiliations:** a Center for Reproductive Medicine of the Second Affiliated Hospital, Center for Regeneration and Cell Therapy of Zhejiang University-University of Edinburgh Institute (ZJU-UoE Institute), Zhejiang University School of Medicine, Zhejiang University, Hangzhou, Zhejiang 310003, China; b Zhejiang University-University of Edinburgh Institute (ZJU-UoE Institute), Zhejiang University School of Medicine, Zhejiang University, Hangzhou, Zhejiang 310003, China; c College of Materials and Chemical Engineering, Minjiang University, Fuzhou, Fujian 350108, China; d National Key Laboratory of Biobased Transportation Fuel Technology, Haining, Zhejiang 314400, China

**Keywords:** Cell-fate transition, Embryonic stem cell, Epigenetic modification, m^6^A modification, Post-transcriptional regulation

## Abstract

As the most prevalent and reversible internal epigenetic modification in eukaryotic mRNAs, *N*^6^-methyladenosine (m^6^A) post-transcriptionally regulates the processing and metabolism of mRNAs involved in diverse biological processes. m^6^A modification is regulated by m^6^A writers, erasers, and readers. Emerging evidence suggests that m^6^A modification plays essential roles in modulating the cell-fate transition of embryonic stem cells. Mechanistic investigation of embryonic stem cell maintenance and differentiation is critical for understanding early embryonic development, which is also the premise for the application of embryonic stem cells in regenerative medicine. This review highlights the current knowledge of m^6^A modification and its essential regulatory contribution to the cell fate transition of mouse and human embryonic stem cells.

## Introduction

RNA modifications play critical roles in epigenetic regulation of gene expression. More than 150 types of post-transcriptional modifications in RNAs have been characterized.^1^ Since first discovered in the 1970s, *N*^6^-methyladenosine (m^6^A) represents the most prevalent internal mRNA modification in eukaryotic cells, accounting for approximately 50% of total methylated ribonucleotides.^2^, ^3^, ^4^, ^5^ The profiling of m^6^A in mammalian cells for the whole transcriptome was first captured in 2012, with the invention of m^6^A antibody-based RNA-immunoprecipitation strategies including m^6^A-seq^6^ and MeRIP-seq.^7^ m^6^A is predominately enriched in 3′ untranslated regions (3′UTRs) and close to stop codons, a feature that is highly conserved across different species.^6^, ^7^, ^8^ In addition, m^6^A also occurs in precursor mRNAs, long non-coding RNAs, and ribosomal RNAs, indicating the broad participation of m^6^A modification in RNA metabolism.^9^, ^10^, ^11^

m^6^A modification was considered static and immutable until the discovery of fat mass and obesity-associated protein (FTO) as the first genuine m^6^A demethylase that reverses the *N*^6^-methyladenosine to adenosine.^12^ Since then, m^6^A modification has been recognized as a dynamic and reversible biological process. This triggers the identification and investigation of important m^6^A regulatory proteins and their biological functions, including writers, erasers, and readers for m^6^A modification. “Writers” are the methyltransferases that add methyl groups to adenosines in RNAs. “Erasers” are demethylases that remove the m^6^A modification from RNAs. While “readers” are RNA-binding proteins that recognize m^6^A-modified RNAs and trigger diverse downstream effects.^13^ A series of recent studies have shown that these proteins have notable effects on the regulation of mRNA processing and metabolism through m^6^A-mediated pathways, including mRNA splicing, nuclear export, mRNA decay, stabilization, and translation efficiency.^14^, ^15^, ^16^, ^17^

Furthermore, m^6^A modification has been discovered to be involved in a wide range of developmental processes including embryogenesis,^18^ neurogenesis,^19^ and diseases such as cancers,^20^ Alzheimer's disease,^21^ and atherosclerosis.^22^ During embryogenesis, dramatic epigenetic changes in the zygote facilitate cellular division and differentiation to form pluripotent embryonic cells. These cells subsequently undergo lineage specification to generate three germ layers for building the embryos. Recently, accumulating evidence suggests that m^6^A modification also plays a crucial role in modulating the cell fate transition of embryonic stem cells (ESCs),^23^^,^^24^ highlighting the importance of epitranscriptomic regulation in setting and/or resetting cell fates during embryonic development. This review focuses on the regulation of m^6^A modification and its potential roles in modulating the pluripotent states of mouse and human ESCs.

## The dynamic regulation of m^6^A modification

**Figure 1:**
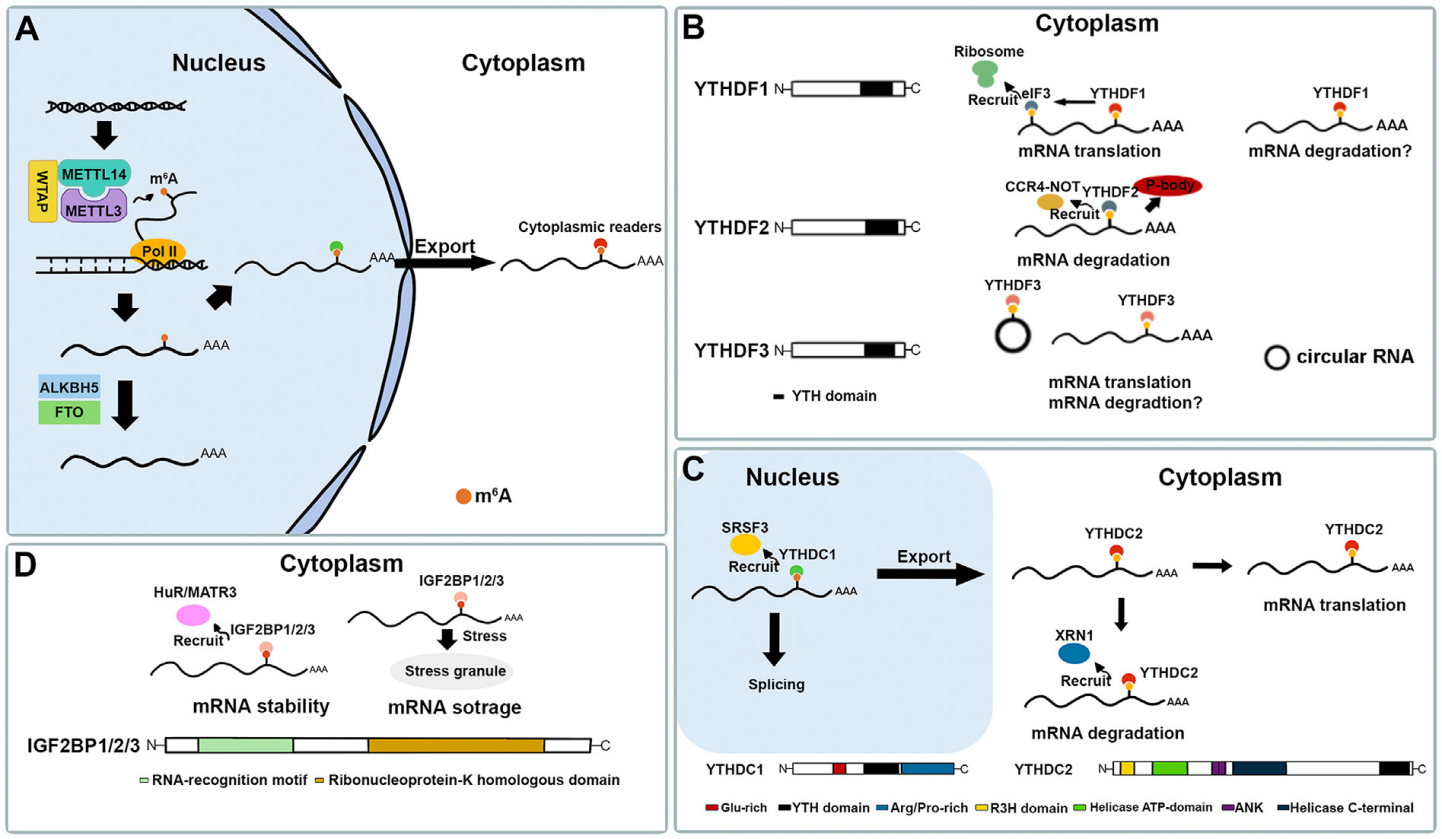
Overview of m^6^A writers, erasers, and readers. **(A)** The m^6^A writers and erasers. In the nucleus, the m^6^A methyltransferase complex (writers) is composed of the core protein METTL3 and its partners WTAP and METTL14. They function together to add methyl groups to mRNAs. In contrast, m^6^A demethylases (erasers) such as ALKBH5 and FTO eliminate m^6^A modification. Readers in the nucleus and the cytoplasm recognize the m^6^A site and play critical roles in mRNA processing and metabolism. **(B)** The m^6^A readers of YTHDF family. YTHDF1 interacts with eIF3 to enhance the mRNA translation efficiency by recruiting ribosomes. YTHDF2 is responsible for promoting mRNA degradation by recruiting CCR4-NOT and target mRNAs to processing bodies. Similar to YTHDF1, YTHDF3 facilitates the translation of both linear and circular mRNAs. **(C)** The m^6^A readers of YTHDC family. In the nucleus, YTHDC1 affects the splicing and export of mRNAs by recruiting SRSF3. In the cytoplasm, YTHDC2 recruits XRN1 to promote the decay of mRNAs or enhance the mRNA translation via the helicase domain. **(D)** The m^6^A readers of IGF2BP proteins. Ribonucleoprotein K homology domain is responsible for RNA binding. IGF2BPs increase mRNA stability by recruiting HuR and MATR3 proteins and preventing the degradation of mRNAs. Additionally, they also regulate mRNA storage under stress conditions.

m^6^A writers, erasers, and readers together compose the m^6^A regulatory machinery. The writers and erasers cooperate to dynamically control the balance of m^6^A abundance, while m^6^A readers recognize m^6^A-modified sites to trigger the downstream effects on target mRNAs (Fig. 1).

## m^6^A writers

The writer complex for m^6^A in mRNAs was initially identified and isolated in 1994 which included two components, methyltransferase component A (MT-A) and B (MT-B).^25^^,^^26^ MT-A plays a key role in methylation while MT-B may exert the regulatory functions. One of the MT-A subunits, MT-A70, named methyltransferase-like protein 3 (METTL3), contains the S-adenosylmethionine-binding site and is the core subunit to catalyze m^6^A formation.^13^^,^^25^^,^^26^ METTL3 is subsequently found to form a stable heterodimer along with one of its homologues, METTL14 (Fig. 1A). Although METTL14 is an inactive methyltransferase, it plays critical roles in maintaining the stability of the complex. Through the binding of METTL14, the methyltransferase activity of METTL3 is strongly increased, highlighting the structural and functional contributions of each protein in the complex.^8^^,^^15^

Later on, WT1-associated protein (WTAP), the most well-studied m^6^A writer-complex regulator, has been reported to be required for the accumulation of METTL3 and METTL14 in nuclear speckles^8^^,^^27^ (Fig. 1A). There are many other regulators in the writer complex, such as vir-like m^6^A methyltransferase associated protein (VIRMA/KIAA1429),^28^ zinc finger CCCH-type containing 13 (ZC3H13),^29^ Cbl photo oncogene like 1 (CBLL-1/HAKAI),^30^ and RNA binding motif protein 15/15B (RBM15/15B).^31^ These regulators are involved in the formation, stabilization, and 3′ UTR enrichment of m^6^A modification.^13^ However, the precise mechanisms underlying the roles of these regulators in different biological contexts remain largely elusive.

## m^6^A erasers

Compared with m^6^A writers, m^6^A erasers are less diverse. Until now, only two enzymes, namely FTO and alkB homolog 5 (ALKBH5), have been identified to mediate m^6^A demethylation (Fig. 1A). In 2011, FTO, a member of the AlkB family, was discovered as the first m^6^A eraser.^12^ The depletion of FTO in HeLa and 293FT cells significantly increased the m^6^A abundance in mRNAs, indicating that m^6^A modification is under dynamic regulation.^12^ Interestingly, another study found that the preferential substrate of FTO is *N*^6^,2′-*O*-dimethyladenosine (m^6^Am) instead of m^6^A.^32^ Importantly, FTO has been reported to regulate m^6^A demethylation in long-interspersed element-1 (LINE1) in mouse embryonic cells, which in turn shapes chromatin state leading to the precise control of gene expression.^33^ In 2013, another m^6^A eraser, ALKBH5, which is specifically enriched in testis, was found to exhibit the ability to demethylate m^6^A modification.^34^ Importantly, ALKBH5 regulates the differentiation of human pluripotent stem cells towards pancreatic lineage in an m^6^A-dependent manner.^35^ These studies highlight the critical roles that the reversible m^6^A modification plays during embryonic development.

## m^6^A readers

As executors of the m^6^A modification, m^6^A readers bind to m^6^A sites to mediate subsequent reaction cascades (Fig. 1). Different m^6^A readers have different functions, and even a single m^6^A reader may trigger different cascade reactions, leading to different fates of the target RNAs. Due to the widespread use of methylated probe pull-down and quantitative mass spectrometry assays, multiple RNA binding proteins were identified as m^6^A readers. Currently, there are mainly two families of m^6^A readers, the YTH domain-containing proteins^36^ and the insulin-like growth factor 2 mRNA-binding protein (IGF2BP) family members.^16^

YTH domain-containing proteins include the YTHDF family, YTHDC1, and YTHDC2 (Fig. 1B, C), which contain the YTH domain that directly recognizes and binds to m^6^A sites. The YTHDF family contains three proteins: YTHDF1, YTHDF2 and YTHDF3. The analysis via an RNA affinity chromatography approach combined with mass spectrometry identified YTHDF2/3 as m^6^A binding proteins. Furthermore, YTHDF1 as an m^6^A reader is found to promote protein synthesis by interacting with translation machinery.^6^^,^^17^ YTHDF1/2/3 share similar sequences and structures (Fig. 1B), and they are all cytoplasmic proteins involved in enhancing m^6^A-modified mRNA phase separation.^37^ However, the specific role of each reader in different biological contexts is still under debate (Fig. 1B). Conventionally, these three proteins have different functions on m^6^A-modified mRNAs. YTHDF1 is reported to enhance the translation efficiency of m^6^A-modified mRNAs,^17^ and YTHDF3 is also shown to regulate both RNA degradation and translation efficiency.^38^ Conversely, YTHDF2 is found to be responsible for the m^6^A-mediated decay by facilitating the localization of RNAs to decay sites.^39^ A following study reveals that YTHDF2 promotes RNA degradation mainly via CCR4–NOT deadenylase complex.^40^ However, some studies demonstrated that YTHDF1/3 have a similar function as YTHDF2 in promoting mRNA degradation, regardless of translation efficiency.^41^^,^^42^ Furthermore, a recent study proposes that YTHDFs have a combined action in mediating the m^6^A-modified mRNA decay,^43^ in contrast to the previous model that each YTHDF mediates different functions by parallelly binding to different mRNA subsets.^44^ These studies emphasize the complex and context-dependent functions of m^6^A readers and indicate that further investigation is required to understand the different roles of YTHDF family proteins in different biological processes.

YTHDC1 is identified as an m^6^A reader in the nucleus to regulate alternative splicing and nuclear export of mRNAs (Fig. 1C), mainly by interacting with the splicing mediator SRSF3 and nuclear export adaptor, respectively.^14^^,^^45^ Moreover, recent studies have revealed that after m^6^A recognition, YTHDC1 plays a critical role in either transcriptional activation or repression through various mechanisms, including the reprogramming of histone modifications,^46^, ^47^, ^48^, ^49^ regulation of enhancer RNAs,^50^ and interaction with long noncoding RNAs (lncRNAs),^31^ highlighting the importance and complexity of RNA-chromatin cross-talk. Thus, YTHDC1 has multiple roles in responding to m^6^A modification. As for YTHDC2, the binding force between its YTH domain and m^6^A-modified RNAs is weaker, compared with that of YTHDC1.^51^ It shares a domain similar to RNA helicases (*e.g.*, DHX29). The main function of YTHDC2 is to promote translation efficiency in testes, safeguarding the process of spermatogenesis.^29^^,^^52^ Besides, YTHDC2 may mediate RNA degradation by interacting with the 5′–3′ exoribonuclease XRN1^53^ (Fig. 1C). These studies further emphasize the context-dependent functions of m^6^A readers in different biological processes.

IGF2BP proteins are a group of relatively newly defined m^6^A readers (Fig. 1D). They are enriched in the m^6^A consensus “GGAC” motif via K homology domains.^16^ IGF2BPs enhance the translation, stability, and storage of their target mRNAs. Specifically, IGF2BPs protect target mRNAs from being degraded in processing bodies by recruiting mRNA-stabilizing proteins such as ELAV-like RNA-binding protein 1 (ELAVL1/HuR) and matrin 3 (MATR3), which is critical for mRNA stability. For mRNA storage, IGF2BPs translocate target mRNAs to stress granules under stress conditions.^16^ Recent studies have also revealed the important roles of IGF2BP proteins in mediating the progression of many types of cancer in a m^6^A-dependent manner, such as bladder cancer,^54^ glioblastoma,^55^ and acute myeloid leukemia.^56^ Expectedly, more m^6^A-binding proteins are identified to expand the reservoir of m^6^A readers for executing different post-transcriptional regulation of RNAs, such as heterogeneous nuclear ribonucleoprotein (HNRNP) family,^57^ fragile X mental-retardation protein (FMR1),^58^^,^^59^ and proline-rich coiled-coil 2A.^60^^,^^61^

Collectively, the cooperation of writers and erasers makes m^6^A methylation a dynamic and regulated process. Different readers that harbor different structures and cellular locations influence almost all aspects of RNA metabolism. As the understanding of the dynamic process of m^6^A methylation expands, it is important to comprehend the physiological implications of this RNA modification, especially during embryogenesis and in the context of ESCs where regulation at the RNA level plays crucial roles.^62^

## Mouse and human ESCs in different pluripotent states

**Figure 2:**
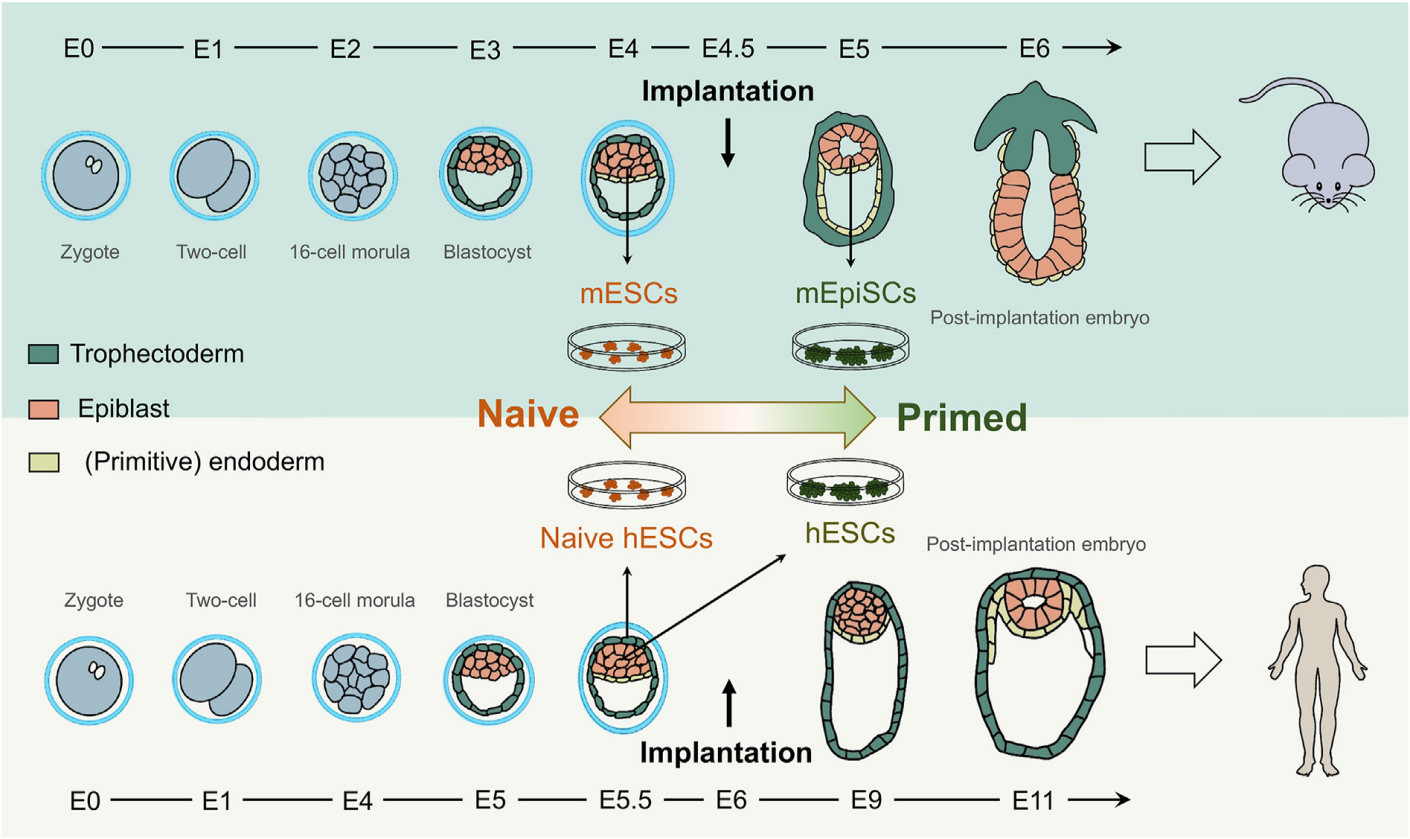
Early embryonic development in mice and humans. The early embryogenesis of mice and humans shares a relatively similar process but with different timelines. The zygote divides for several rounds to form the morula at embryonic day 2 (E2) in mice and E4 in humans. Subsequently, cells undergo the first lineage specification to form the trophectoderm and inner cell mass at E3 in mice and E5 in humans. Before implantation, cells in the inner cell mass further differentiate into a layer of the primitive endoderm and epiblast cells. Implantation occurs at around E4.5 in mice and E6 in humans. After implantation, epiblast cells undergo gastrulation to form three germ layers, which finally constitute the whole body of the embryo. In mice, cells can be derived from the epiblasts in the pre-implanted embryo and cultured *in vitro* as mESCs at a naïve pluripotent state. Cells derived from the post-implanted epiblasts are called mEpiSCs at primed pluripotent state. In humans, hESCs are derived from the pre-implanted epiblasts, which surprisingly show primed pluripotency similar to mEpiSCs when cultured in conventional media. Recently, multiple strategies have also been applied to capture hESCs with naïve pluripotency.

Embryonic cells at around the time of implantation are pluripotent, holding the potential to differentiate into all the cells in the embryo proper.^63^ In mice, embryonic cells from the inner cell mass and the pre-implantation epiblasts can be maintained *in vitro* indefinitely as mouse embryonic stem cells (mESCs) in the pluripotent state called naïve state^64^, ^65^, ^66^ (Fig. 2). Through blastocyst injection, mESCs are able to constitute a high proportion of chimeric mice and can be transmitted to the germline.^67^ Epiblast cells derived from post-implantation mouse embryos are pluripotent and can be induced to differentiate into cells of the three germ layers, however, without the ability to give rise to chimeric mice. These post-implantation epiblast-derived stem cells (mEpiSCs) are distinct from mESCs in epigenetic state and gene expression patterns.^68^^,^^69^ Therefore, the pluripotent state of mEpiSCs is defined as the primed state, since they are more primed for differentiation.^65^

Extensive research has also been carried out to obtain human ESCs (hESCs) from pre-implantation epiblasts of the human embryos, leading to the first established hESC lines in 1998^70^ (Fig. 2). The features of hESCs are found to be more similar to those of mEpiSCs at the primed state rather than mESCs at the naïve state.^64^^,^^71^ Because of the limitations in using primed ESCs as a model to study the mechanisms of early embryonic development, it is essential to culture hESCs in an earlier stage, such as the naïve state. Recently, a wide range of protocols have been established to maintain hESCs with similar but not identical features to mESCs in a naïve state of pluripotency.^72^ The development of mESCs and hESCs has captured most of the molecular signatures of the early mouse and human embryogenesis, respectively (Fig. 2). This greatly facilitates the *in vitro* investigation into the early events of development, including the roles of m^6^A modifications. Because of the significant differences in mESCs and hESCs, the roles of m^6^A in mESCs and hESCs will be discussed separately.

## The functions of m^6^A modification in mESCs

Accumulating studies have shown that m^6^A modification regulates the pluripotent state and preserves the ESC identity by influencing the mRNA metabolism in mESCs (Table 1). Currently, m^6^A writers have been widely studied for their roles in these processes, while the investigation of m^6^A erasers and readers in mESCs is relatively limited.

**Table 1:** The phenotypes of the depletion of m^6^A-related components in human embryonic stem cells (hESCs) and mouse embryonic stem cells (mESCs).

		hESCs	mESCs
Writers	METTL3	*METTL3* knockdown hESCs show impaired differentiation and blocked neuroectoderm differentiation.^24^^,^^77^	*Mettl3* knockout mESCs show enhanced self-renewal and impaired differentiation.^23^^,^^24^
	METTL14	*METTL14* knockdown hESCs show enhanced self-renewal and blocked neuroectoderm differentiation.^77^	*Mettl14* knockout/knockdown mESCs show enhanced self-renewal, impaired differentiation, and further embryonic lethality in gastrulation.^15^^,^^84^^,^^91^
	METTL16	Unknown	*Mettl16* knockout leads to reduced target mRNA levels in 16-cell embryos and mediates transcriptome dysregulation and further developmental disorder in ∼64-cell blastocysts.^92^
	WTAP	*WTAP* knockout hESCs show unaffected pluripotency and blocked neuroectoderm differentiation.^77^	*Wtap* knockout mESCs show defective endoderm and mesoderm differentiation, leading to defective egg-cylinder formation at the gastrulation stage and early death at E10.5^93^; *Wtap* knockdown mESCs show impaired self-renewal and trigger differentiation.^81^
	KIAA1429	Unknown	*Kiaa1429* depletion in oocytes results in infertility.^94^
	RBM15/15B	Unknown	Unknown
	ZC3H13	Unknown	*Zc3h13* knockout mESCs show impaired self-renewal and trigger differentiation.^73^
	CBLL1	Unknown	Unknown
Erasers	FTO	No dramatic phenotype for *FTO* knockout hESCs^95^	*Fto* knockout mESCs up-regulate two cell-like state-related genes, impair self-renewal, and trigger differentiation.^33^ Elevated levels of FTO protein show maintained stem cell pluripotency.^86^
	ALKBH5	*ALKBH5* overexpression remarkably blocks cardiomyocyte differentiation of hESCs.^78^	Unknown
Readers	YTHDC1	Unknown	*Ythdc1* knockout increases the expression of retrotransposons to induce two cell-like state transitions.^48^^,^^49^
	YTHDC2	Unknown	Unknown
	HNRNPC	Unknown	Unknown
	hnRNPA2B1	*hnRNPA2B1* knockdown decreases the expression of pluripotency genes and increases the expression of differentiation genes of three germ layers.^96^	*hnRNPA2B1* knockdown mESCs show impaired pluripotency and self-renewal ability in blastocysts.^97^
	YTHDF1	unknown	Single knockout of *Ythdf1/2/3* does not affect the self-renewal ability and expression of pluripotency genes, while triple-knockout shows poor differentiation ability and a hyper-naïve state in mESCs.^42^*Ythdf1* knockout mESCs impair cardiomyocyte differentiation, while *Ythdf3* depletion mESCs facilitate cardiomyocyte differentiation.^98^
	YTHDF2	unknown	
	YTHDF3	unknown	
	IGF2BP1	*IGF2BP1* knockdown decreases cell–cell adherence, disrupts actin cytoskeleton, and reduces cell proliferation.^99^	Unknown
	IGF2BP2	Unknown	Unknown
	IGF2BP3	Unknown	Unknown
	FMR1	Unknown	Unknown
	LRPPRC	Unknown	Unknown
	ELAVL1	Unknown	unknown

## Writers in mESCs

**Figure 3:**
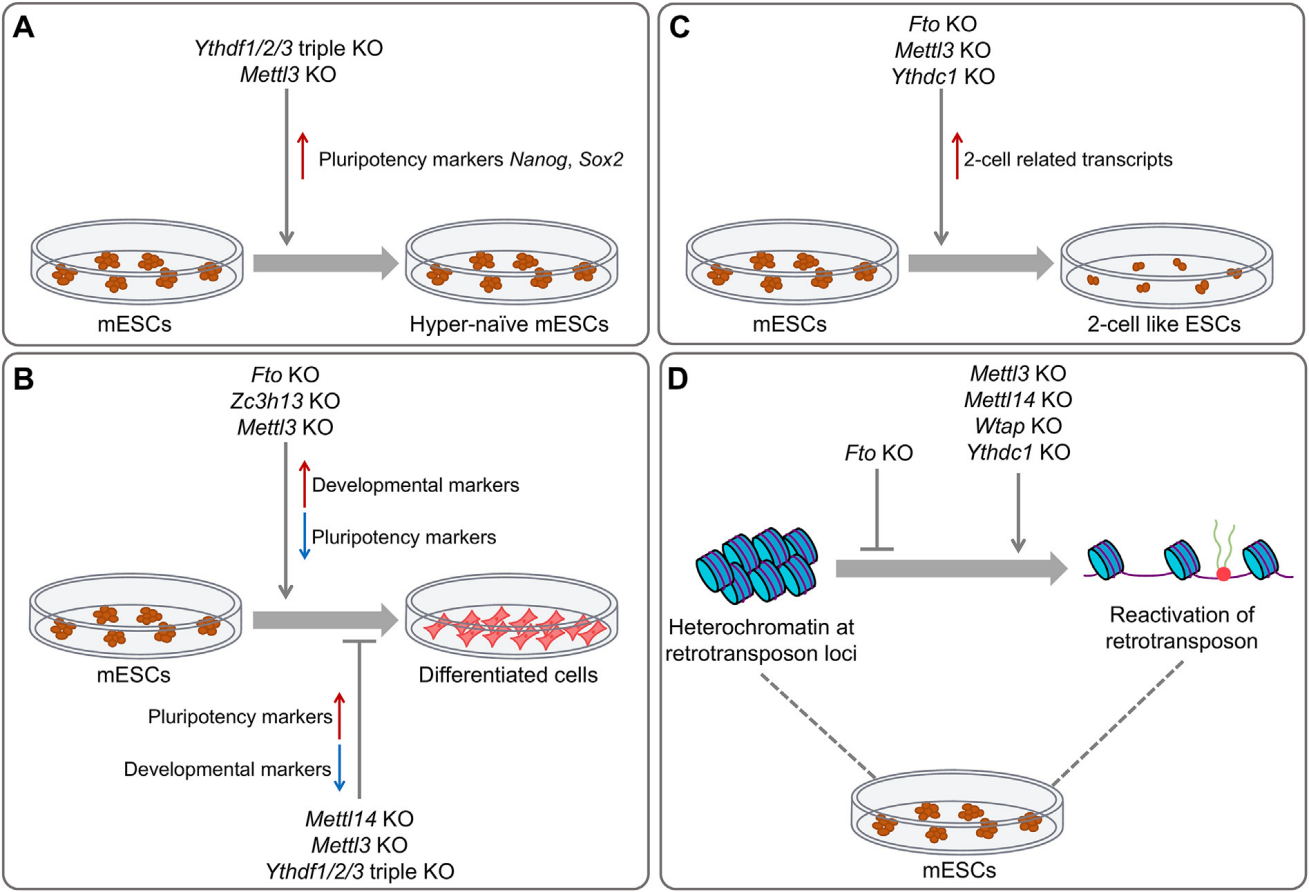
Functions of m^6^A-related proteins in mESCs. **(A)***Mettl3* knockout and triple-knockout of *Ythdf1/2/3* prolonged the expression of pluripotency markers (*e.g.*, *Nanog* and *Sox2*), resulting in a hyper-naïve phenotype. **(B)** The controversial effect of m^6^A writers on mESC differentiation. Knockdown of *Mettl3* or *Zc3h13* leads to impaired differentiation capacity, while knockout of *Mettl3* or *Mettl14* increases the differentiation capacity. **(C)** Knockout of m^6^A eraser *Fto* leads to impaired differentiation and triple-knockout of *Ythdf1/2/3* results in increased differentiation capacity. Knockout of *Mettl3*, *Fto*, and *Ythdc1* leads to a transcriptomic 2-cell like transition. **(D)** Knockout of *Mettl3*, *Mettl14*, and *Wtap* increases the expression of retrotransposons (*e.g.*, LINEs and IAPs), while knockout of *Fto* decreases LINE1 abundance. KO, knockout; KD, knockdown.

The expression of m^6^A writers starts at the very beginning of embryogenesis.^18^ One of the most well-studied functions of m^6^A writers during early embryogenesis is to deposit m^6^A on pluripotency-related transcripts in mESCs, which influences the stem cell fate decisions^15^^,^^23^^,^^24^ (Fig. 3).

To be more specific, m^6^A modification is believed to regulate the exit of pluripotency in mESCs. It was first reported that m^6^A modification was deposited on core pluripotency transcripts in mESCs to facilitate mRNA degradation.^24^ Subsequent research confirmed that the *Mettl3* depletion reduced the global m^6^A level in mESCs and in mouse embryos at the peri-implantation stage. The prolonged expression time of pluripotency genes such as *Nanog*, and the impaired cell differentiation in the *Mettl3* mutant both *in vitro* and *in vivo*, suggest that m^6^A is critical in regulating the pluripotent states in embryonic cells during development^23^ (Fig. 3A). Similarly, depletion of *Mettl14*, the structural subunit in the m^6^A writer complex, resulted in aberrant cell differentiation and embryonic lethality^18^ (Fig. 3B).

However, there are some different observations for the functions of m^6^A writers in regulating mESCs. It was illustrated that *Mettl3* knockdown promoted cellular differentiation in mESCs by inhibiting the expression of pluripotency-related genes (*e.g.*, *Nanog* and *Sox2*) and up-regulating the expression of developmental markers (*e.g.*, *Sox17*)^15^ (Fig. 3B). In addition, the knockdown of *Zc3h13*, which serves as an anchor to help nuclear localization of ZC3H13-WTAP-Virilizer-Hakai complex, impaired stem cell self-renewal and stimulated differentiation^73^ (Fig. 3B). These controversial observations may be due to different culture conditions, different ESC lines, and different techniques applied to either knock out or knock down the key components of m^6^A writers.

In addition to the sophisticated regulation of the maintenance and differentiation of mESCs, m^6^A writer METTL3 has also been found to be involved in the modulation of heterochromatin, whose integrity is critical for retrotransposon repression (Fig. 3D). Through its catalytic activity, METTL3 establishes m^6^A modifications at transcripts of retrotransposon RNAs including the LINE1 family and the endogenous retroviral elements. These modifications provide binding sites for m^6^A reader YTHDC1, which in turn leads to RNA degradation and/or facilitates the formation of heterochromatin marks at the corresponding loci.^48^^,^^49^^,^^74^, ^75^, ^76^ It has been found that *Mettl3* knockout abolishes m^6^A modifications on 25 of the 45 m^6^A-modified retrotransposon RNAs, and up-regulates a group of retrotransposons that are repressed by SET domain bifurcated histone lysine methyltransferase 1 (SETDB1)-dependent H3K9me3.^48^^,^^75^ The decrease of m^6^A caused by *Mettl3* knockout also results in increased stability of LINE1 RNAs, which facilitates the open chromatin state and downstream transcription.^74^ In addition to its catalytic activity, METTL3 also recruits repressive histone modifiers to regulate the integrity of intracisternal A particle (IAP) heterochromatin, inhibiting the transcription of IAP RNAs. METTL3 predominantly localizes in IAP loci. In conjugation with YTHDC1, it recruits SETDB1 and its cofactor TRIM28 to deposit heterochromatin mark H3K9me3 at IAP loci and inhibit its transcription.^48^^,^^75^ Considering that endogenous retroviral elements and LINE1 are activated specifically at the 2-cell (2C) stage, the altered integrity of heterochromatin may be a causal factor of the transcriptional 2C state transition induced by *Mettl3* knockout^48^ (Fig. 3C).

In addition to METTL3, other writers are also involved in the regulation of retrotransposons. For example, through an unbiased genome-scale CRISPR knockout screen, it has been found that the depletion of METTL3-METTL14, as well as their accessory subunits WTAP and ZC3H13, increases the RNA abundance of IAPs (Fig. 3D). It may be achieved by interfering with the YTHDFs-mediated degradation of these IAP RNAs.^76^ Taken together, all these studies emphasize the critical functions of m^6^A writers in regulating the maintenance and differentiation of mESCs through different mechanisms.

## Erasers in mESCs

Erasers cooperate with writers to regulate the RNA metabolism dynamically and rapidly. Since the activity of m^6^A erasing is limited to specific tissues or conditions, the role of erasers is considered narrow.^13^ However, recent research reveals that m^6^A eraser FTO mediates m^6^A demethylation of LINE1 RNA, modulating its abundance and corresponding chromatin accessibility, which therefore regulates the transcription of LINE1-containing genes. Knockout of *Fto* increases LINE1 degradation and a reduction of its transcription, leading to the down-regulation of LINE1 expression (Fig. 3D). *Fto* knockout also leads to the up-regulation of 2C-related genes, dysregulation of the cell cycle, impairment of self-renewal, increased differentiation capacity, and decreased pluripotency of mESCs (Fig. 3B, C). These phenotypic changes are similar to those occurring after LINE1 antisense oligo treatment.^33^ Therefore, FTO plays a key role in regulating early embryonic development through the FTO-LINE1 RNA axis.

## Readers in mESCs

m^6^A readers are executors of m^6^A functions. Currently, readers including YTHDFs and YTHDC1 have been identified to be involved in the maintenance and differentiation of mESCs (Fig. 3), while the role of IGF2BPs in mESCs has not been demonstrated.

Recent studies have shown that YTHDFs play an essential role in regulating mESC differentiation potential redundantly. Neither knockout of a specific *Ythdf* reader nor triple-knockout of *Ythdf1/2/3* down-regulates the self-renewal ability and the expression of pluripotency markers in mESCs. However, while wild-type (WT) and single-knockout mESCs can differentiate properly, triple-knockout mESCs show a poor differentiation ability and a hyper-naïve pluripotency phenotype during the generation of teratoma and embryoid bodies (Fig. 3A, B). In the triple-knockout embryoid bodies, differentiation markers (*e.g.*, *Fgf5*, *Gata6*, and *Sox17*) were barely expressed, whereas pluripotency markers (*e.g.*, *Nanog*, *Rex1*, and *Sox2*) were adequately expressed. In addition, triple-knockout of *Ythdf1/2/3* increases the half-life of m^6^A-modified mRNAs, indicating their roles in mRNA degradation. Surprisingly, overexpression of any of the three *YTHDF* readers alone is sufficient to rescue the proper differentiation of mESCs, which supports the functional redundancy of YTHDF1/2/3 in mESCs.^42^

Despite the redundant effect of YTHDFs on mESC self-renewal and differentiation, YTHDF1/3 may have different functions in mESCs, for which YTHDF2 is not involved. In mESCs with *Ythdf1* knockout, *Ythdf3* knockout, or triple-knockout, but not *Ythdf2* knockout, 2C-related transcripts are shown to be up-regulated, indicating the potential roles of YTHDF1/3 in promoting the degradation of mRNAs of the 2C-related genes. However, rather than an enrichment for 2C-related genes, binding profile analysis in mESCs reveals enrichment of YTHDF1 and YTHDF3 targets for blastocyte genes. A potential explanation of this phenomenon is that typically 2C-related genes are not expressed in mESCs, whereas blastocyst genes are. Thus, the regulatory role of YTHDF1 and YTHDF3 should be investigated in depth in 2C stage embryos to further understand the functions of m^6^A readers in regulating mouse embryogenesis.

In addition to YTHDFs, accumulating evidence also supports that nuclear protein YTHDC1 plays an essential role in repressing the expression of retrotransposons, facilitating the maintenance of mESC identity. Specifically, once bound with m^6^A labeled retrotransposon RNAs (such as IAP and LINE1), YTHDC1 recruits SETDB1 to deposit H3K9me3. The resulting closed chromatin conformation inhibits the transcription of retrotransposons at the corresponding loci.^48^ Conditional knockout of *Ythdc1* increases the expression of retrotransposons and induces a 2C-like transition in mESCs (Fig. 3C, D).^48^^,^^49^ This transition is dependent on Dux, a master inducer of the 2C-like transition, whose locus is occupied by LINE1 RNA based on the result of ChIRP-seq and GRID-seq. This Dux-dependent transition is further confirmed by the fact that *Dux* knockout was sufficient to block the 2C-like transition induced by *Ythdc1* deletion. In addition, *Dux*-knockout mESCs retain the ability to reactivate many 2C-related retrotransposons in the context of *Ythdc1* knockout, indicating that their YTHDC1-mediated repression is independent of *Dux*-regulated 2C-like transition.^48^

The YTHDC1-mediated repression mechanism is also supported by other studies where different chromatin modifiers are recruited.^49^^,^^75^ Detailed analysis showed that YTHDC1 recognizes a group of LINE1 RNAs with METTL3-insensitive m^6^A sites (not affected by *Mettl3* knockout) and facilitates the formation of the LINE1-nucleolin-KAP1 complex. This complex promotes the recruitment of KAP1 and facilitates the deposition of repressive H3K9me3 at targets of the LINE1 scaffold including 2C-related retrotransposons.^49^ Additionally, METTL3-mediated m^6^A modification provides a binding site for YTHDC1, which in turn leads to more recruitment of METTL3 to IAP loci. As mentioned, in conjunction with YTHDC1, METTL3 recruits SETDB1 and TRIM28 (the co-factor of SETDB1) to deposit repressive H3K9me3 at IAP loci and decrease the transcription of IAP RNAs.^75^

In addition to recruiting repressive chromatin modification proteins to inhibit retrotransposon transcription, YTHDC1 also regulates the stability of retrotransposon-derived RNAs. For instance, YTHDC1 recognizes the m^6^A-modified LINE1 RNAs and promotes their degradation through interaction with components of the nuclear exosome targeting complex that is responsible for the degradation of specific nuclear RNAs.^74^ Therefore, by regulating the decay of m^6^A-modified retrotransposons and heterochromatin silencing, YTHDC1 plays an essential role in preventing abnormal activation of retrotransposons, thus ensuring the programmed cell fate transition during embryonic development.

## The functions of m^6^A modification in hESCs

Studies on the functional roles of m^6^A in hESCs have been initiated around the same time as those in mESCs (Table 1). There are several conserved features of m^6^A modification in mESCs and hESCs, such as the consensus motif of RRACH, as well as the enrichment of m^6^A at 3′UTR, near stop codons, or long internal exons in both species.^7^^,^^77^ Even though mESCs and hESCs are in different pluripotent states and cultured in different conditions, comparative epitranscriptomic analysis has identified 3609 conserved m^6^A-modified transcripts (69.4%) between them, which reveals the conservation of these modification events during evolution.^24^

**Figure 4:**
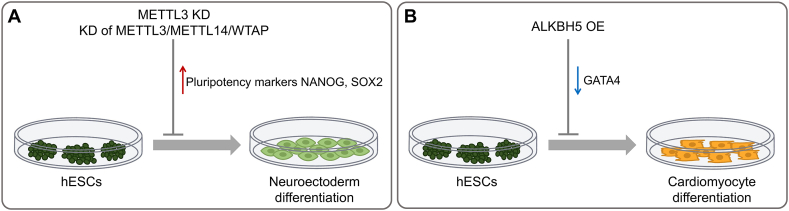
Functions of m^6^A writers and erasers in hESC. **(A)** Both *METTL3* knockdown and multiple knockdowns of *METTL3/METTL14/WTAP* cause the prolonged expression of pluripotency-related genes including *NANOG* and *SOX2*, impairing the neuroectoderm differentiation. **(B)** Overexpression of *ALKBH5* results in the down-regulation of *GATA4*, an important transcription factor for cardiac lineage specification, which in turn impairs hESC cardiac commitment. KD, knockdown; OE, overexpression.

As for m^6^A-related proteins, recent studies have identified the critical roles of m^6^A writers and erasers in hESCs (Fig. 4), while the functions of readers remain largely unknown. In hESCs, m^6^A writers exhibit similar functions as in mESCs to deposit m^6^A methylation in the mRNAs of core pluripotency factors, which results in their degradation upon differentiation.^24^ Knockdown of *METTL3* significantly reduces m^6^A deposition in hESCs, leading to prolonged expression of *NANOG* and *SOX2* during hESC differentiation, and impairing the exit from pluripotency^24^ (Fig. 4A). Remarkably, it has been found that TGF-β signaling regulates hESC pluripotency via SMAD2/3 by interacting with m^6^A machinery.^77^ In the presence of activin-NODAL signaling, SMAD2/3 activates the transcription of pluripotency factors. Meanwhile, it also facilitates the recruitment of the METTL3-METTL14-WTAP complex to promote m^6^A deposition on the downstream transcripts, leading to their timely degradation. Such negative feedback maintains the subtle balance of the abundance of the pluripotency-related transcript. Thus, upon loss of activin-NODAL signaling, these m^6^A-containing pluripotency transcripts undergo rapid down-regulation, leading to the timely exit from pluripotency and toward neuroectoderm specification.^77^

m^6^A erasers also play critical roles in hESC fate decisions. Overexpression of m^6^A demethylase ALKBH5 significantly blocked cardiomyocyte differentiation of hESCs^78^ (Fig. 4B). Mechanistically, ALKBH5-mediated m^6^A demethylation elevates the level of lysine demethylase 5B and decreases the level of a histone lysine methyltransferase complex subunit retinoblastoma binding protein 5 by altering the stability of their mRNAs, which impairs the H3K4me3 at the promoter region of *GATA4*. Subsequently, the impaired transcription of *GATA4* inhibits cardiomyocyte lineage commitment of hESCs.^78^

Although the critical roles of m^6^A in regulating the fate specification of hESCs have been studied as mentioned above, the global alteration of methylation levels at thousands of sites in these experiments limits the investigation of individual m^6^A sites within a transcript of interest. To understand the function of m^6^A modification on specific mRNAs, a targeted RNA m^6^A erasure system was developed to remove m^6^A methylation site-specifically. It was achieved by coupling the RNA-targeting capability of CRISPR-dCas13a with the catalytic ALK domain of ALKBH5. In this way, the dCas13a-ALKBH5 was guided to the specific mRNAs by gRNAs to remove the m^6^A modification. Targeted demethylation of *SOX2* mRNA at A1398, which prolonged *SOX2* mRNA level, promoted ectodermal but inhibited endodermal and mesodermal differentiation of hESCs, again highlighting the importance of m^6^A in regulating hESC pluripotency.^79^

## The regulation of m^6^A machinery in ESCs

**Figure 5:**
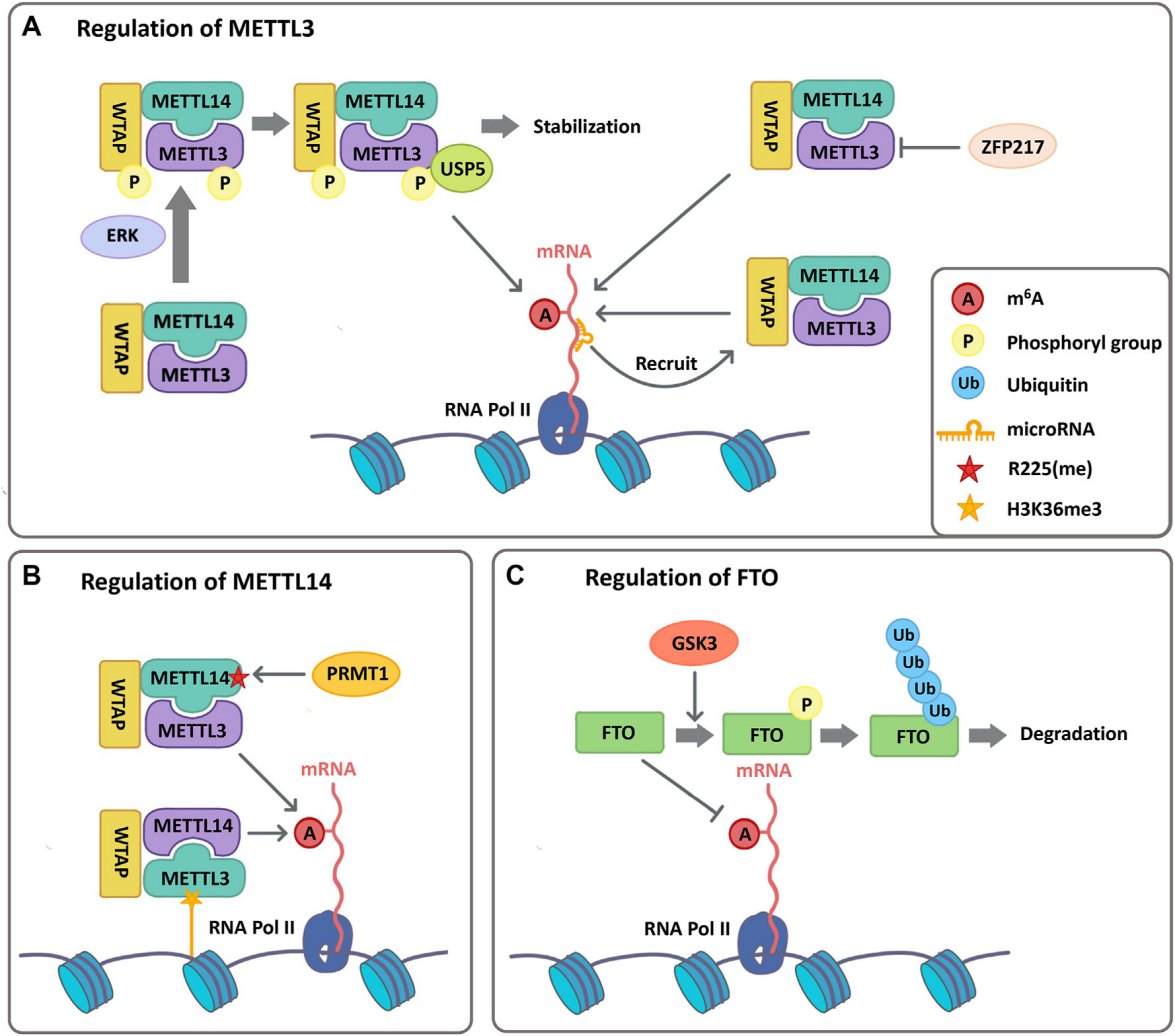
The regulation of m^6^A machinery in embryonic stem cells. **(A)** Regulation of METTL3. ERK pathway-regulated phosphorylation on METTL3 and WTAP triggers the USP5-mediated deubiquitination, which stabilizes m^6^A MTC and promotes m^6^A modifications. ZFP217 sequesters METTL3 to decrease m^6^A methylation in mRNAs. MicroRNAs bind to unmethylated sequences of mRNAs and recruit METTL3 to the nuclear speckles, promoting the *de novo* m^6^A deposition. **(B)** Regulation on METTL14. PRMT1 mediates arginine methylation in METTL14 R255, enhancing the interaction of METTL3/METTL14 with WTAP and MTC binding to RNA substrates, which promotes m^6^A modifications in mRNAs. H3K36me3 binds to METT14 directly and promotes MTC interaction with RNA Pol II, thus depositing m^6^A co-transcriptionally. **(C)** Regulation of FTO. GSK-3 mediates the phosphorylation of FTO, followed by polyubiquitination and degradation, which increases m^6^A modifications in mRNAs. ERK, extracellular signal regulated kinase; USP5, ubiquitin specific peptidase 5; MTC, methyltransferase complex; ZFP217, zinc-finger protein 217; PRMT1, protein arginine N-methyltransferase 1; R255(me), methylated arginine 255; RNA Pol II, RNA polymerase II; GSK-3, glycogen synthase kinase-3.

Both transitions of stem cell fate and the maintenance of ESC identity require temporal and spatial regulation of gene expression. To ensure the stringent gene expression pattern, regulators are needed to control m^6^A machinery precisely. Accordingly, studies have revealed diverse regulations of m^6^A writers and erasers in mESCs, which affect m^6^A abundance in transcripts and determine cell fates in different stages (Fig. 5).

## The regulation of METTL3

As a catalytic protein in the m^6^A methyltransferase complex, METTL3 plays a core role in m^6^A deposition, and its upstream regulation is widely studied, which includes direct inhibition by other proteins, phosphorylation modification, and microRNA-mediated recruitment (Fig. 5A). ZFP217 is proved to balance self-renewal and differentiation of mESCs by restraining METTL3 activity.^80^ Comparing the phenotypes between *Zfp217* knockdown and WT mESCs, ZFP217 protein is shown to play a critical role in the maintenance of mESC self-renewal by sequestering METTL3 and subsequently reducing the global level of m^6^A modification. As mESCs progress to differentiation, the expression of ZFP217 declines rapidly, allowing m^6^A to be deposited to mRNAs for pluripotency factors via METTL3 to trigger their degradation.^80^

Another regulation mechanism is the ERK pathway-mediated phosphorylation of METTL3, which is followed by ubiquitin-specific peptidase 5 (USP5)-catalytic deubiquitination. As a result, the METTL3-METTL14-WTAP complex is stabilized, permitting the decay of the mRNAs of pluripotency-related genes and thus allowing proper mESC differentiation.^81^

Additionally, microRNAs were discovered to modulate the binding of METTL3 to mRNAs, inducing *de novo* m^6^A deposition in HeLa cells. This modulation is achieved via a sequence pairing mechanism. When microRNAs recognize and bind to the unmethylated sequences of mRNAs, they may recruit METTL3 to the nuclear speckles and facilitate the *de novo* deposition of m^6^A. Deletion of *Dicer*, an important enzyme in microRNA production, significantly blocks the subcellular localization of METTL3 at nuclear speckles. Further, an increased m^6^A level regulated by microRNAs was proved to actively promote cell reprogramming efficiency from mouse embryonic fibroblasts to induced pluripotent stem cells.^82^ However, how it is related to ESC fate decisions remains to be determined.

## The regulation of METTL14

Unlike METTL3, METTL14 has no enzymatic activity and serves as a structural scaffold to stabilize the methyltransferase complex. Since the stability of the complex is proven to increase the methylation activity of METTL3, the regulation of METTL14 is also important. Histone modification-mediated recruitment and arginine methylation have been reported to regulate METTL14 in mESCs^83^ (Fig. 5B). H3K36me3, a transcriptional activation marker, is recently found to recruit METTL14 to deposit m^6^A co-transcriptionally. In this process, METTL14 recognizes and interacts with H3K36me3, promoting the binding between m^6^A methyltransferase complex and transcribing nascent mRNAs. H3K36me3 modification is crucial for the normal exit from pluripotency in mESCs, as its depletion leads to a higher level of pluripotency transcripts (such as *Oct4* and *Nanog*) and increased stemness.^84^ It is hypothesized that arginine methylation of METTL14 may enhance the binding of METTL14 and H3K36me3 modification through lipid–lipid phase separation *in vivo*. In turn, H3K36me3 may promote arginine methylation of METTL14, permitting accumulated METTL14 with high activity and increased m^6^A modification.^83^

Arginine methylation of METTL14 by PRMT1 is also indispensable for pluripotency exit in mESCs.^83^^,^^85^ Without arginine methylation in R255 of METTL14, the global level of m^6^A decreased significantly, blocking the decay of pluripotency-related mRNAs and further impairing endoderm differentiation.^85^ Mechanistically, arginine methylation in R255 of METTL14 not only enhances the interactions among proteins in the m^6^A methyltransferase complex but also promotes the binding between this complex to substrate RNAs. Improved interactions have been detected between the m^6^A methyltransferase complex and RNA substrates *in vitro*, which likely increases the complex activity. Moreover, arginine methylation facilitates the interactions between METTL14 and RNA polymerase II during transcription.^83^^,^^85^

## The regulation of m^6^A erasers

Unlike writers, there is still limited information about the regulation of m^6^A erasers. Currently, the only known regulation is the phosphorylation of FTO, which is mediated by GSK-3, leading to the polyubiquitination and further degradation of FTO in mESCs (Fig. 5C). With the double knockout of GSK-3, the level of FTO proteins increased greatly while the global m^6^A level reduced by 50%. Subsequently, the decay of the pluripotency-related mRNA was impaired and mESCs without GSK-3 exhibited prolonged pluripotency.^86^

Notably, m^6^A modification-mediated regulation intermingles with other post-transcriptional regulation, as well as transcriptional regulation at the chromatin level, leading to the complex mechanisms governing the gene expression that ensure the self-renewal and differentiation of ESCs. Despite the crucial roles of m^6^A modification, the underlying functions and molecular mechanisms governing the regulation of ESCs and embryogenesis are still unknown. There are two main reasons. One is that deletion of m^6^A-related genes may result in early embryonic lethality, and the other is that the techniques for analyzing m^6^A profiles in developing embryos remain limited.

## Perspectives

Humans and mice were diverged approximately 60 million years ago, they exhibit species-specific differences in early embryogenesis.^64^^,^^87^ Conventional mESCs and hESCs are in naïve and primed pluripotent states,^64^ respectively, further complicating the divergent regulatory roles for embryonic development. Nonetheless, the m^6^A writers, erasers, and readers are expressed and play key roles in regulating the self-renewal and differentiation of ESCs in both humans and mice, although the detailed mechanisms may vary. Basically, m^6^A modification-mediated regulation is involved in both human and mouse ESCs by facilitating the decay and/or stabilization of pluripotency and/or differentiation transcripts, permitting cell fate regulation during development. Notably, m^6^A modification is involved in epigenetic regulation at the chromatin level by modulating histone modifications^48^^,^^75^ and DNA methylation,^88^ opening a new avenue for understanding the cross-talk of gene regulation at the transcriptional level and post-transcriptional level. This is important for ensuring the cell fate transition and determination for precise and programmed development. A better understanding of the m^6^A modification-mediated regulation for ESC maintenance and differentiation will help the application of ESCs for regenerative medicine by facilitating pure and functional differentiated cells.

Based on these discoveries of m^6^A modification in ESCs, new questions emerge and require further investigation. What are the functions of m^6^A writers/erasers/readers in primed mEpiSCs and in naïve hESCs? Are there new writers/erasers/readers in ESCs compared with other cell types? Whether m^6^A modification is also involved in regulating retrotransposons in hESCs? To what extent the discoveries based on ESCs could be applied to *in vivo* embryogenesis? How is m^6^A modification-encoded epigenetic information interpreted to regulate cell fate during differentiation? Previously, studies have focused on the composition of m^6^A regulators, especially m^6^A writers and erasers, and how they determine the m^6^A patterns in different cell types. However, emerging studies also revealed the importance of m^6^A readers, as they directly interpret the epigenetic information encoded by m^6^A modification and they trigger diverse cascades leading to different fates of the target mRNAs. Therefore, considering the regulation of m^6^A formation, how desired subsets of transcripts are labeled with m^6^A, and how specific readers recognize and mediate particular functions are highlighted issues in the future.

With the development of new technology, these questions may be answered in the future. For example, some newly developed techniques for m^6^A profiling, such as m^6^A-SAC-seq^89^ and ULI-MeRIP–seq,^90^ are able to detect m^6^A modification at single-base resolution with a small amount of RNA, overcoming the limited resources of human embryos and achieving m^6^A epitranscriptiome with better resolution. Additionally, the combination of the CRISPR system and m^6^A regulators, such as the targeted RNA m^6^A erasure system that can eliminate specific m^6^A modifications, makes the study of m^6^A functions more precisely.^79^ As m^6^A studies progress to the site-specific era, a deeper insight into the epigenetic modeling in embryogenesis will be provided, advancing our understanding of developmental diseases and stimulating new stem cell-based therapies.

## Author contributions

JZ, LT, and DC design the structure of the review. JZ, LT, YL, XL, JW, RL, ZZ, YC, YC, and DC drafted the manuscript. JZ, LT, ZZ, YC, YC, and DC revised the manuscript. XL, YL, and DC drafted and revised the figures. All authors read and approved the final manuscript.

## Conflict of interests

The authors have no competing interests to declare.

## Funding

This work was supported by the 10.13039/501100001809National Natural Science Foundation of China (No. 32270835 to DC) and the 10.13039/501100004731Zhejiang Natural Science Foundation (No. Z22C129553 to DC).
